# A conditional-lethal vaccinia virus mutant demonstrates that the I7L gene product is required for virion morphogenesis

**DOI:** 10.1186/1743-422X-2-4

**Published:** 2005-02-08

**Authors:** Chelsea M Byrd, Dennis E Hruby

**Affiliations:** 1 Molecular and Cellular Biology Program, Oregon State University, 220 Nash Hall, Corvallis, Oregon, 97331 USA; 2 Department of Microbiology, Oregon State University, 220 Nash Hall, Corvallis, Oregon, 97331 USA

## Abstract

A conditional-lethal recombinant virus was constructed in which the expression of the vaccinia virus I7L gene is under the control of the tetracycline operator/repressor system. In the absence of I7L expression, processing of the major VV core proteins is inhibited and electron microscopy reveals defects in virion morphogenesis subsequent to the formation of immature virion particles but prior to core condensation. Plasmid-borne I7L is capable of rescuing the growth of this virus and rescue is optimal when the I7L gene is expressed using the authentic I7L promoter. Taken together, these data suggest that correct temporal expression of the VV I7L cysteine proteinase is required for core protein maturation, virion assembly and production of infectious progeny.

## 

Proteolytic cleavage of precursor proteins is an essential process in the life cycle of many viruses, including vaccinia virus (VV). The cysteine proteinase encoded by the VV I7L gene, was originally identified based on a sequence comparison with the African Swine Fever virus proteinase and an ubiquitin-like proteinase in yeast [1,2]. We have previously shown through *trans *processing assays that the I7L gene product is capable of cleaving the core protein precursors p4a, p4b, and p25K at conserved AG/X sites and have used reverse genetics to identify active site residues [3,4]. To determine the role that the I7L proteinase plays in the VV replication cycle, we report here the construction and *in vivo *analysis of a VV mutant in which the expression of the I7L gene can be conditionally regulated.

While this work was in progress, Ansarah-Sobrinho and Moss [5] published a report demonstrating that the I7L proteinase, in an inducible mutant virus regulated by the *lac *operator and driven off of the T7 promoter, was responsible for cleaving the A17L membrane protein as well as the L4R core protein precursor. In this work, we show that I7L proteinase, in a different inducible mutant virus, this one regulated by the tetracycline (TET) operator/repressor system and driven off of the I7L native promoter, is responsible for cleaving the other core protein precursors (p4a and p4b). We also demonstrate that expression of the I7L gene from its native promoter appears to be important for optimal viral assembly and replication.

To investigate the role of the I7L proteinase in the viral life cycle, an inducible mutant virus was constructed in which the expression of the I7L gene could be regulated by the presence or absence of TET using the components of the bacterial tetracycline operon [6,7]. This system has been shown to be successful in the regulation of the vaccinia virus G1L [8,9] and A14L [10] genes. A plasmid was constructed containing the tetO just upstream of the I7L open reading frame (ORF) in order to regulate expression of I7L proteinase with TET in the presence of a tetracycline repressor (TetR). Also included was the genomic DNA sequence from 250 bp upstream of the I7L ORF, to include the native promoter, and to aid in homologous recombination. This plasmid was used to create the recombinant virus vtetOI7L using the transient dominant selection method [11]. A commercially available cell line, T-Rex-293 (Invitrogen), expressing the TetR was used to regulate the expression of the I7L gene from the infecting recombinant virus. This conditional-lethal expression system has recently been used to show that the enzymatic activity of the VV G1L metalloproteinase is essential for viral replication [9].

The conditional-lethal phenotype of the recombinant virus was shown by plaque assay (Fig. 1), in which the formation of plaques from vtetOI7L is dependent on the presence of TET, while the wild-type virus is unaffected by either the presence or absence of TET. To determine the optimum TET concentration required for replication of vtetOI7L, TREx-293 cells were infected with vtetOI7L in the presence of varying concentrations of TET, harvested 24 h later, and the titer determined on BSC_40 _cells [12]. A 2-log increase in viral yield was observed with 1 μg/ml TET (data not shown). To confirm that expression of the I7L gene was essential for viral replication, TREx-293 cells were infected with vtetOI7L at a multiplicity of infection (MOI) of 0.1, 0.5, 5, or 10 in the presence or absence of TET, harvested 24 h later, and the titer of the virus infected cell lysates determined on BSC_40 _cells. At an MOI of 0.1 or 0.5 there was an average reduction of 99.1% of infectious virus particles (Fig. 2). At an MOI of 5 there was an average reduction of 95.7%, and at an MOI of 10 there was an average reduction of 90.3% (Fig. 2). This multiplicity-dependent breakthrough of viral replication is likely due to gene copy overwhelming the amount of TetR being expressed by the TREx-293 cell line.

**Figure 1:**
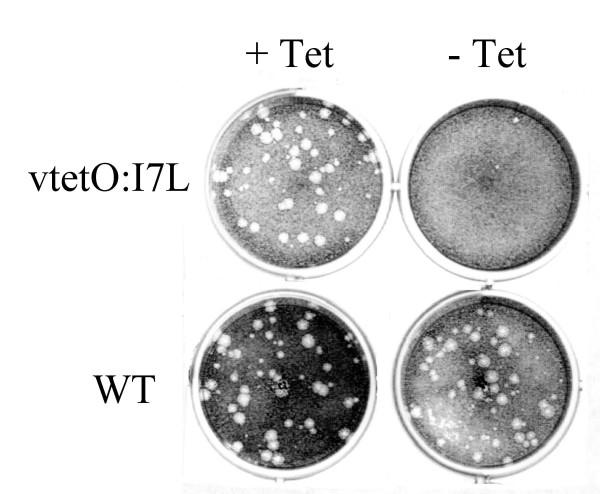
**Effect of TET on plaque formation. **TREx-293 cells were infected with vtetOI7L or wild-type virus in the presence or absence of 1 μg/ml TET and harvested 24 hpi. BSC_40 _cells were then infected and stained with crystal violet 48 hpi.

**Figure 2:**
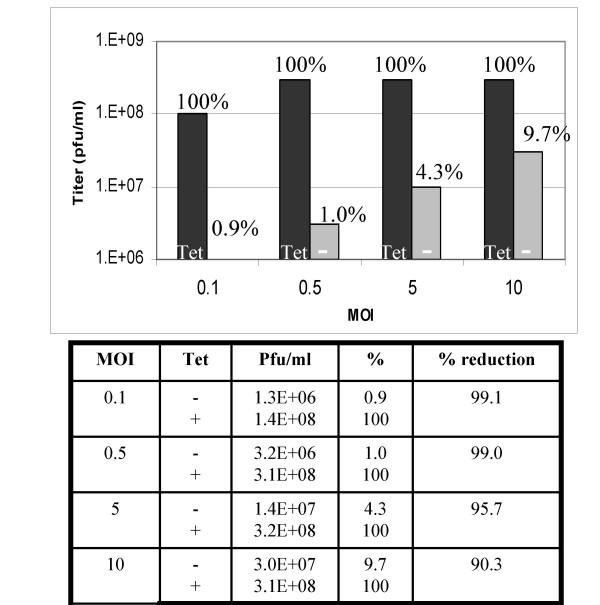
**Effect of TET on viral replication and rescue of the vtetOI7L mutant. **TREx-293 cells were infected with vtetOI7L in the absence (-) or presence of 1 μg/ml TET at an MOI of 0.1, 0.5, 5, or 10. Infected cells were harvested 24 hpi and titrated on BSC_40 _cells.

To test whether the insertion of the TET operator just upstream of the I7L ORF had an effect on the viral growth kinetics, a one-step growth curve was conducted. TREx-293 cells were infected with wild type virus or vtetOI7L in the presence or absence of TET and infected cell lysates were harvested at the indicated times and the titer determined on BSC_40 _cells (Fig. 3A). In the presence of TET, the recombinant virus grew to the same yield and with the same kinetics as wild type virus while in the absence of TET the production of infectious virus was much lower indicating that the presence of the TET operator did not have an effect on the growth kinetics of the inducible mutant virus.

**Figure 3:**
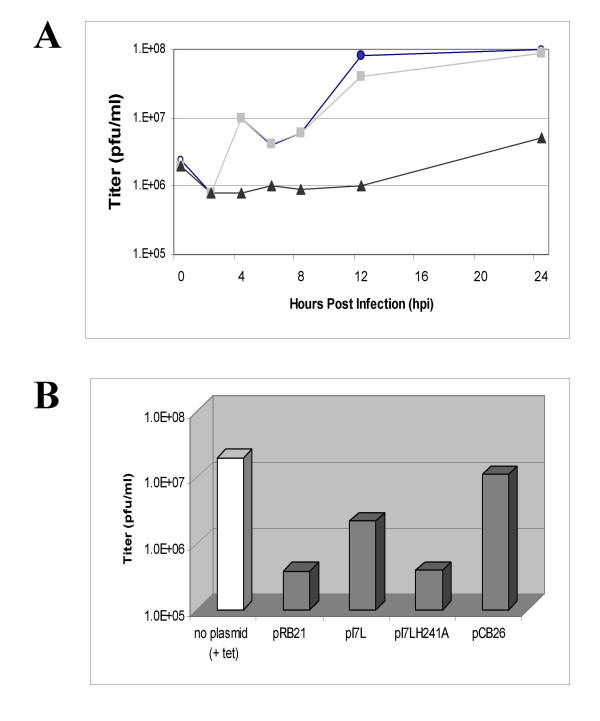
**Panel A: One step growth curve. **TREx-293 cells were infected with wild-type virus (circle) or vtetOI7L in the presence (square) or absence (triangle) of 1 μg/ml TET. Infected cells were harvested at the indicated times and the titer determined on BSC_40 _cells. **Panel B: Rescue of replication. **TREx-293 cells were infected with vtetOI7L and transfected with either vector alone (pRB21), plasmid with wild-type I7L driven off of a synthetic early/late promoter (pI7L), plasmid with mutant I7L, mutated in the putative active site, driven off of a synthetic early/late promoter (pI7LH241A), or wild-type I7L driven off of its native promoter (pCB26) in the absence of TET. Infected cells were harvested 24 hpi and the titer determined on BSC_40 _cells. Transfection of plasmid borne wild-type I7L but not of mutant I7L or vector alone partially rescued the replication of vtetOI7L.

To demonstrate that the replication defect of the vtetOI7L mutant virus in the absence of TET was due to the I7L gene we tested whether viral replication could be rescued by the introduction of a plasmid-borne I7L gene. TREx-293 cells in 6-well plates were transfected with 1.8 μg of plasmid DNA (containing either no insert, a wild type I7L gene under the control of the synthetic early-late promoter, a I7L gene with the catalytic His241 mutated to Ala, or the I7L gene under the control of its native promoter) and infected with vtetOI7L at an MOI of 0.2 plaque-forming units per cell in the absence of TET. Cells were harvested 24 hours post infection (hpi) and the titer determined on BSC_40 _cells. As an additional control, TREx-293 cells were mock transfected and infected with vtetOI7L in the presence of 1 μg/ml TET to compare growth conditions. A partial rescue of viral replication was observed when cells were transfected with the I7L gene under the control of the synthetic early/late promoter, but not when cells were transfected with plasmid alone or with a mutant I7L gene (Fig. 3B). This was an approximate 5-fold increase in virus replication compared to the pRB21 or pI7LH241A transfected controls. When the I7L gene was driven off of its own promoter in pCB26 and transfected in, there was a much higher level of rescue (Fig. 3B), suggesting that the timing and amount of I7L gene expression has important implications for the viral life cycle.

We have previously shown through transient expression assays that the I7L proteinase is capable of cleaving the p4b, p4a, and p25k core protein precursors [3,4] which are products of the A3L, A10L, and L4R open reading frames respectively. Here we were interested to see whether the I7L proteinase in the conditional lethal mutant system was also capable of cleaving these proteins in the presence but not the absence of TET. First, to see whether I7L protein was expressed at the same time from the mutant virus as from the wild type virus, TREx-293 cells were infected in the presence of TET and cells harvested at various time points. Proteins in the crude cell extracts were separated by SDS-PAGE and detected by Western blot with anti-I7L antisera. I7L enzyme from both viruses appeared at late times after infection, around 8 hpi and increased as time progressed (data not shown). To determine the effect of TET on I7L protein expression, cells were infected and treated with 0 to 5 μg/ml TET. After 6 h, the infected cells were labeled with 60 μCi/ml ^35^S-met and harvested after 24 h. Extracts were immunoprecipitated with I7L antisera and protein detected by autoradiography. With wild type virus, I7L protein was expressed at each TET concentration (data not shown). However, in the mutant virus, expression of I7L enzyme was repressed in the absence of TET and increased with the addition of TET.

To determine the effect of TET concentration on p4b core protein precursor processing, cells were infected in the presence of 0 to 5 μg/ml TET, harvested 24 hpi, and the extracts immunoblotted with anti-4b antisera. With wild type virus p4b was processed at each TET concentration as expected, however with the mutant virus, p4b processing was repressed in the absence of TET (data not shown). The slight processing in the absence of TET is likely due to slight leak-through of I7L gene expression in this system. The same results were seen for the processing of p4a, with processing in each of the wild type virus lanes, repressed processing with the mutant in the absence of TET and increased processing in the presence of TET (data not shown). Kane and Shuman [13] have previously shown that I7L protein is located in the virus core. To verify that the I7L protein from the inducible mutant was localized correctly, purified virions were treated with DTT and NP-40 to separate the envelope fraction from the core fraction and protein from each sample was separated by SDS-PAGE and detected by Western blot with anti-I7L antisera. As expected, the I7L enzyme from the inducible mutant was detected in the core sample, as was the wild type virus (data not shown).

The morphogenesis of vtetOI7L under nonpermissive conditions was analyzed via electron microscopy. TREx-293 cells were infected with vtetOI7L at an MOI of 1 in the presence or absence of TET and harvested 24 h later. In the presence of TET, cells contained a variety of both immature and mature forms of the virus (Fig. 4, panels A-C), which were indistinguishable from cells infected with wild type virus (not shown). However, in the absence of TET, no mature virions were observed in any of the infected cells observed. There appeared to be an accumulation of immature viral particles, some with nucleoids, as well as the appearance of crescent shaped particles (Fig. 4, panels D-F), similar to those observed by Ansarah-Sobrinho *et al *[5]. Also observed were numerous dense virus particles. Virion morphogenesis appears to arrest at a stage prior to core condensation. The observation that there is still some processing of p4b in the absence of TET and yet the morphology of the mutant virus in the absence of TET shows only immature virus particles suggests the hypothesis that there is a requirement for the processing threshold of the core protein precursors to be achieved before morphogenesis can proceed.

**Figure 4:**
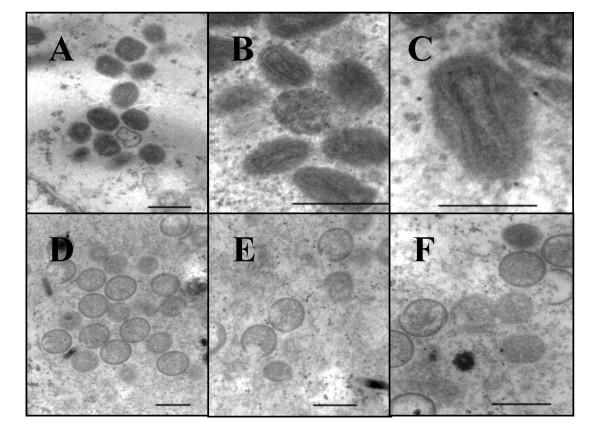
**Electron microscopy of cells infected with vtetOI7L. **TREx-293 cells were infected with vtetOI7L at an MOI of 1 in the presence (panels A, B, and C) of 10 μg/ml TET or in the absence (panels D, E, and F) of TET. Cells were harvested at 24 hpi, immediately fixed and prepared for transmission electron microscopy. The bar in panels A, B, D, E, and F represents 400 nm. The bar in panel C represents 200 nm.

Taken together, the data we have presented here, as well as analysis of the VV G1L conditional lethal mutant [9], suggests a morphogenesis model in which these two putative proteases operate sequentially to regulate assembly. According to this model, if we assume that both I7L and G1L are associated with the immature virus along with the accompanying DNA and other viral proteins, then activation of I7L leads to the process of core protein precursor cleavage and the initiation of core condensation. Following this activity, the activation of G1L completes core condensation and allows progression to the formation of intracellular mature virus. If the activity of the I7L proteinase is blocked, viral morphogenesis arrests prior to core condensation. If the activity of G1L proteinase is blocked, viral morphogenesis arrests at a stage subsequent to this but still prior to complete core condensation. To test this model, it will be of interest to isolate biochemically active I7L and G1L enzymes and determine the series of events that lead to their activation.

## Competing Interests

The author(s) declare that there are no competing interests.

## Authors' contributions

CMB conducted all the experiments and wrote the manuscript. DEH conceived the study, coordinated the research efforts and edited the paper. Both authors read and approved the final manuscript.
